# A potential therapeutic effect of CYP2C8 overexpression on anti-TNF-α activity

**DOI:** 10.3892/ijmm.2014.1844

**Published:** 2014-07-09

**Authors:** WANJUN LIU, BEI WANG, HU DING, DAO WEN WANG, HESONG ZENG

**Affiliations:** The Institute of Hypertension and Department of Internal Medicine, Tongji Hospital, Tongji Medical College, Huazhong University of Science and Technology, Wuhan, Hubei 430030, P.R. China

**Keywords:** CYP2C8, epoxyeicosatrienoic acids, inflammation, reactive oxygen species, nuclear factor-κB, atherosclerosis

## Abstract

Epoxyeicosatrienoic acids (EETs) are generated from arachidonic acid catalysed by cytochrome P450 (CYP) epoxygenases. In addition to regulating vascular tone EETs may alleviate inflammation and ROS. The present study was conducted to determine whether *CYP2C8* gene overexpression was able to increase the level of EETs, and subsequently prevent TNF-α induced inflammation and reactive oxygen species (ROS) in human umbilical vein endothelial cells (HUVECs) and macrophages. Peroxisome proliferator-activated receptor γ (PPARγ) activation, nuclear factor-κB (NF-κB) activation, endothelial nitric oxide synthase (eNOS) activation, gp-91 activation, and inflammatory cytokine expression were detected by western blot analysis or enzyme-linked immunosorbent assay. Intracellular reactive oxygen species (ROS) was measured by flow cytometry, while the migration of vascular smooth muscle cells (VSMCs) was detected by Transwell assay. pCMV-mediated CYP2C8 overexpression and its metabolites, EETs, markedly suppressed TNF-α induced inflammatory cytokines IL-6 and MCP-1 expression via the activation of NF-κB and degradation of IκBα. Moreover, pretreatment with 11,12-EET significantly blocked TNF-α-induced ROS production. CYP2C8-derived EETs also effectively alleviated the migration of VSMCs and improved the function of endothelial cells through the upregulation of eNOS, which was significantly decreased under the stimulation of TNF-α. Furthermore, these protective effects observed were mediated by PPARγ activation. To the best of our knowledge, the results of the present study demonstrated for the first time that CYP2C8-derived EETs exerted antivascular inflammatory and anti-oxidative effects, at least in part, through the activation of PPARγ. Thus, the *CYP2C8* gene may be useful in the prevention and treatment of vascular inflammatory diseases.

## Introduction

Atherosclerosis is a complex pathological process that possesses many features of chronic inflammation (1) and oxidative stress in the vascular wall. It also plays a major role in atherogenesis. Reactive oxygen species (ROS), generated as byproducts of cellular metabolism, elicit numerous effects on cell functions in several cell types such as endothelial cells, macrophages and vascular smooth muscle cells (VSMCs) (2).

In endothelial cells, ROS regulate numerous signaling pathways including those regulating cell growth, proliferation, vaso-relaxation, inflammatory responses, barrier function and vascular remodeling (3). The vascular ROS production by NADPH oxidase or mitochondria is markedly enhanced in atherosclerotic arteries, which in turn limits the activity of nitric oxide (NO), thereby producing endothelial dysfunction (4). A number of *in vitro* and *in vivo* studies have demonstrated a critical role for ROS or enzyme systems involved in ROS production, including endothelial NO synthases, xanthine oxidase, enzymes of the respiratory chain, cytochrome P450 monooxygenases and NAD(P)H oxidase in the vasculature (5). Upregulation of the NAD(P)H oxidase subunits gp91phox and Nox4 increases intracellular oxidative stress in macrophages and non-phagocytic vascular cells of human coronary atherosclerosis, respectively (6). Furthermore, the endothelial cell responds to various proinflammatory mediators such as oxLDL. oxLDL has been previously shown to upregulate the expression of MCP-1 via activation of ROS and nuclear factor (NF)-κB (7).

In macrophages, a recent study showed that the CD14/TLR4 (a Toll-like receptor 4)/MD-2 complex interacts with mmLDL, inducing cytoskeletal rearrangements and the secretion of macrophage inflammatory protein-2, MCP-1, tumor necrosis factor-α (TNF-α) and interleukin-6 (8,9) via ROS generation from spleen tyrosine kinase/Nox2 signaling (10). In addition, NF-κB, the most well-known redox-dependent transcriptional factors, regulates a number of genes involved in inflammatory responses in macrophages (11).

In VSMCs, ROS mediates various functions including growth, migration, matrix regulation, inflammation and contraction (12) which are critical factors in the progression and complications of atherosclerosis. In addition, in VSMCs, ROS also mediate inflammation, e.g., MCP-1 expression via TNF-α (13).

The cytokine TNF-α, characterized as a potent pro-inflammatory cytokine, induces oxidative stress in cells and increases intracellular ROS generation (14,15). It also leads to the activation of NF-κB. However, antioxidants have been shown to scavenge intracellular ROS production and block the NF-κB activation (16). These results suggest that the suppression of ROS-dependent intracellular signaling may be an effective strategy for inflammatory vascular diseases.

Epoxyeicosatrienoic acids (EETs) are synthesized predominantly by the epoxygenases of the CYP2 family, including the 2C and 2J classes. CYP2C and CYP2J are mainly expressed in epithelial, endothelial, and smooth muscle cells, as well as cardiomyocytes, autonomic ganglion cells, and islet cells in the heart, vessel, kidney, lung and pancreas (17–20). Specifically, CYP2C8 is expressed mainly in the endothelium. EETs possess a number of biological effects in the cardiovascular and renal systems, including anti-inflammatory (17) and angiogenic (21) effects on endothelial cells, and inhibition of vascular smooth muscle cell migration (22). EETs have recently been reported to attenuate ROS (23). However, how CYP2C8-derived EETs affect ROS signaling pathways that lead to inflammation and atherosclerosis remains to be determined. The focus of the present study was CYP2C8 and its capacity to elucidate how the arachidonic acid metabolites, EETs, attenuate TNF-α induced inflammation through ROS in vascular endothelial cells and macrophages and improve endothelial function and provide new insight into how CYP2C8-derived EETs ameliorate vascular inflammatory diseases such as atherosclerosis.

## Materials and methods

### Materials

Chemicals and reagents were obtained as follows: Dulbecco’s modified Eagle’s medium (DMEM) and fetal bovine serum (FBS) were purchased from HyClone Laboratories, Inc. (Logan, UT, USA); HUVECs, VSMCs, macrophages cell lines and 2′,7′-dichlorodihydrofluorescein diacetate (HB_2B_DCF-DA) were purchased from Wuhan Boster Biological Technology, Ltd. (Wuhan, China); exogenous EETs and PPARγ-specific inhibitor GW9662 were from Cayman Chemical (Ann Arbor, MI, USA); RPMI-1640 medium and recombinant human TNF-α were from Sigma Chemical, Co. (St. Louis, MO, USA); pCMV-CYP2C8 plasmids from OriGene Technologies, Inc. (Rockville, MD, USA) were introduced into cells using Lipofectamine 2000 (Invitrogen, Carlsbad, CA, USA); antibodies against PPARγ, lamin B1 and nuclear factor κB (NF-κB) were from Santa Cruz Biotechnology, Inc. (Santa Cruz, CA, USA); antibodies against gp-91, CYP2C8 and eNOS were from Cell Signaling Technology (Beverly, MA, USA); antibody against β-actin was from Neomarkers (Fremont, CA, USA). All other reagents were purchased from standard commercial suppliers.

### Cell culture

Human umbilical vein endothelial cells (HUVECs) were maintained in RPMI-1640 medium supplemented with 10% FBS, 100 U/ml penicillin and 100 μg/ml streptomycin at 37°C under 5% CO_2_. Macrophages and VSMCs were maintained in DMEM supplemented with 10% FBS, 100 U/ml penicillin and 100 μg/ml streptomycin at 37°C under 5% CO_2_. HUVECs and macrophages were seeded in a 6-well plate at a density of 3×10^5^ cells/well. After the cells reached 60% confluence and the medium was removed, the cells were transferred to serum-free medium. The HUVECs and macrophages were pretreated with exogenous EETs (1 μM) or transfected with CYP2C8 in advance for 30 min in the presence or absence of the specific PPARγ antagonist GW9662 (1 μM), and subsequently stimulated with TNF-α (10 ng/ml) for the indicated times.

### Cell transfection and expression

The plasmids pCMV-CYP2C8 and pCMV-GFP were obtained from OriGene Technologies Inc. (Rockville). As the density of the cultured cells reached ~50–70%, each group was pretreated with Lipofectamine 2000 and then interfered by adding the pCMV-CYP2C8 and pCMV-GFP plasmids, respectively. After 6 h of transfection, the experimental medium was added to the cells followed by exposure to TNF-α (10 ng/ml) in the absence or presence of GW9662 (1 μM). The efficacy of transfection was obtained by examining the green fluorescence by microscopy (Nikon, Tokyo, Japan).

### Intracellular ROS production assay

Confluent HUVECs in 6-well plates were pretreated with EETs for 1 h. Following removal of the EETs from the wells, the cells were incubated with 20 μM HB_2B_DCF-DA for 30 min and then stimulated with TNF-α (10 ng/ml) for 1 h. The fluorescence intensity was measured at an excitation and emission wavelength of 485 and 530 nm, respectively, using a fluorescence spectrophotometer or a fluorescence microscopy (Nikon). Values for each treatment group were recorded as the mean fluorescence intensity.

### VSMCs Transwell assay

Transwsell 12-well plates were obtained from Costar Corp. (Cambridge, MA, USA). Monolayers of serum-starved adherent cells were trypsinized and cell suspensions were placed in serum-free medium in the upper well of a Transwell filter apparatus. The filter was suspended in a well of a 12-well plate and the lower reservoir was filled with the same medium (no cells) plus added TNF-α. The cells were incubated under basal condition for 12 h. The cells were stained with DAPI (Wuhan Boster Biological Technology) and cells found on the underside of the filter were counted by microscopy (Nikon).

### Western blot analysis

HUVECs and macrophages were pre-treated with exogenous EETs (1 μM) and PPARγ inhibitor GW9662 for 1 h, and then stimulated with or without TNF-α (10 ng/ml). Cultured HUVECs and macrophage were lysed in RIPA buffer containing a mixture of protease inhibitors, and the total protein concentration was determined by protein assay. Proteins (50 μg) from cell lysates were electrophoresed by SDS-PAGE, proteins and nuclear extracts were then transferred to a PVDF membrane (Roche Diagnostics, Mannheim, Germany). The membrane was blocked with blocking buffer (1× TBS, 0.1% Tween-20, 5% non-fat dry milk), and washed and incubated overnight at 4°C with anti-NF-κB p-65 (1:1,000 dilution), anti-IκBα (1:1,000 dilution), anti-eNOS (1:1,000 dilution), anti-gp-91 (1:1,000 dilution), anti-β-actin (1:1,000 dilution) or anti-lamin B-1 (1:1,000 dilution) primary antibodies. The membrane was subsequently washed with TBS-T (10 mmol/l Tris-HCl, 150 mmol/l NaCl, and 0.1% Tween-20) and incubated with horseradish peroxidase-conjugated secondary antibodies at 37°C for 1 h. The immune complex was detected with an enhanced chemiluminescence system (Beyotime Institute of Biotechnology, Jiangsu, China), exposed to X-ray film, and analyzed using ImageJ2x software. β-actin and lamin B-1 were used as an internal control.

### Measurement of MCP-1 and IL-6 level by enzyme-linked immunosorbent assay (ELISA)

The level of MCP-1 and IL-6 in the supernatants was measured using a commercially available kit from Wuhan Boster Biological Technology according to the manufacturer’s instructions. Optical densities were recorded on a universal microplate reader (Bio Tek Instruments, VT, Winooski, USA) at 450 nm.

### Statistical analysis

Data are presented as the mean ± SEM. *In vitro* experiments were performed a total of 4–6 times, and each experiment was carried out in triplicate for each treatment group. Statistical comparisons among groups were performed by the Wilcoxon test, the Student’s t-test or ANOVA as appropriate. In all cases, statistical significance was defined as P<0.05.

## Results

### Transfection with CYP2C8 attenuated TNF-α induced inflammation by decreasing the levels of inflammatory factor MCP-1 and IL-6

**Figure 1:**
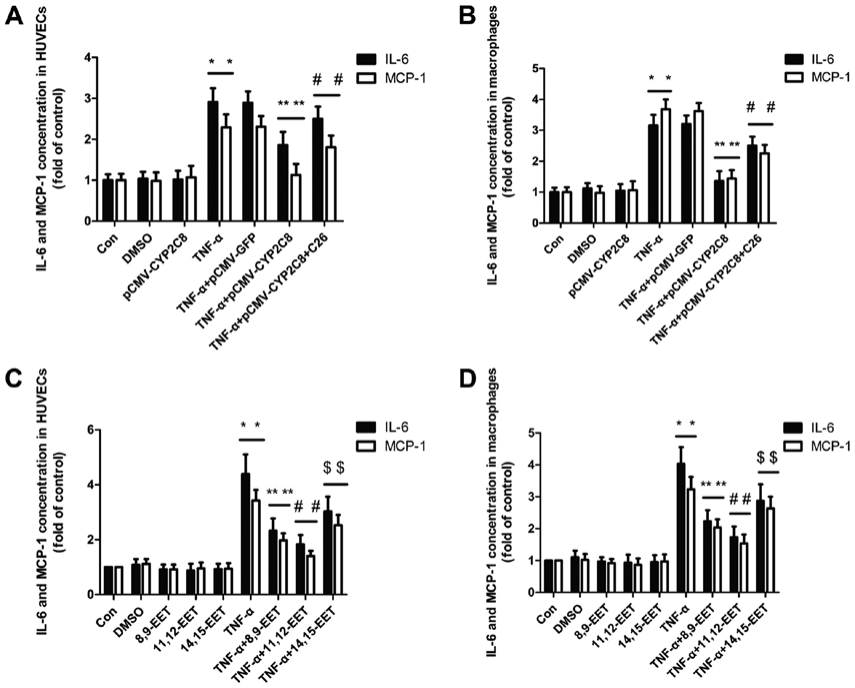
CYP2C8-derived EETs attenuated TNF-α induced inflammation by decreasing the levels of inflammatory factor MCP-1 and IL-6. (A) Primary cultures of HUVECs were transfected with CYP2C8 and administered with CYP2C8 inhibitor C26 for 60 min and stimulated with TNF-α (10 ng/ml) for 24 h. (^*^P<0.05 vs. control group; ^**^P<0.05 vs. TNF-α treatment group; ^#^P<0.05 vs. no inhibitor treatment group). (B) Macrophages were transfected as described in (A). (^*^P<0.05 vs. control group; ^**^P<0.05 vs. TNF-α treatment group; ^#^P<0.05 vs. no inhibitor treatment group). (C) Primary cultures of HUVECs were pre-treated with exogenous 8,9-EET (1 μM), 11,12-EET (1 μM) and 14,15-EET (1 μM), respectively, for 60 min and stimulated with TNF-α (10 ng/ml) for 24 h. (^*^P<0.05 vs. control group; ^**^P<0.05, ^#^P<0.05 and ^$^P<0.05 vs. TNF-α treatment group). (D) Macrophages were pre-treated with exogenous 8,9-EET (1 μM), 11,12-EET (1 μM) and 14,15-EET (1 μM), respectively, for 60 min and stimulated with TNF-α (10 ng/ml) for 24 h. (^*^P<0.05 vs. control group; ^**^P<0.05, ^#^P<0.05 and ^$^P<0.05 vs. TNF-α treatment group). Data are the mean ± SEM of results from at least five independent experiments, each performed in triplicate.

The levels of inflammatory factors MCP-1 and IL-6 were examined in macrophages and HUVECs. pCMV-CYP2C8 delivery led to an abundant CYP2C8 expression and increased EETs generation. ELISA analysis showed that transfection with CYP2C8 markedly suppressed the expression of inflammatory cytokines including interleukin (IL)-6 and MCP-1 induced by TNF-α, which was reversed by CYP2C8 inhibitor C26 (Fig. 1A and B). As shown in Fig. 1C and D, 8,9-EET, 11,12-EET and 14,15-EET markedly reduced the IL-6 and MCP-1 expression induced by TNF-α in HUVECs and macrophages, with 11,12-EET exerting the most significant effect. Therefore, we used 11,12-EET as the representative of EET in the subsequent experiments.

### CYP2C8-derived EETs inhibited TNF-α induced expression of NF-κB through PPARγ in HUVECs and macrophages

**Figure 2:**
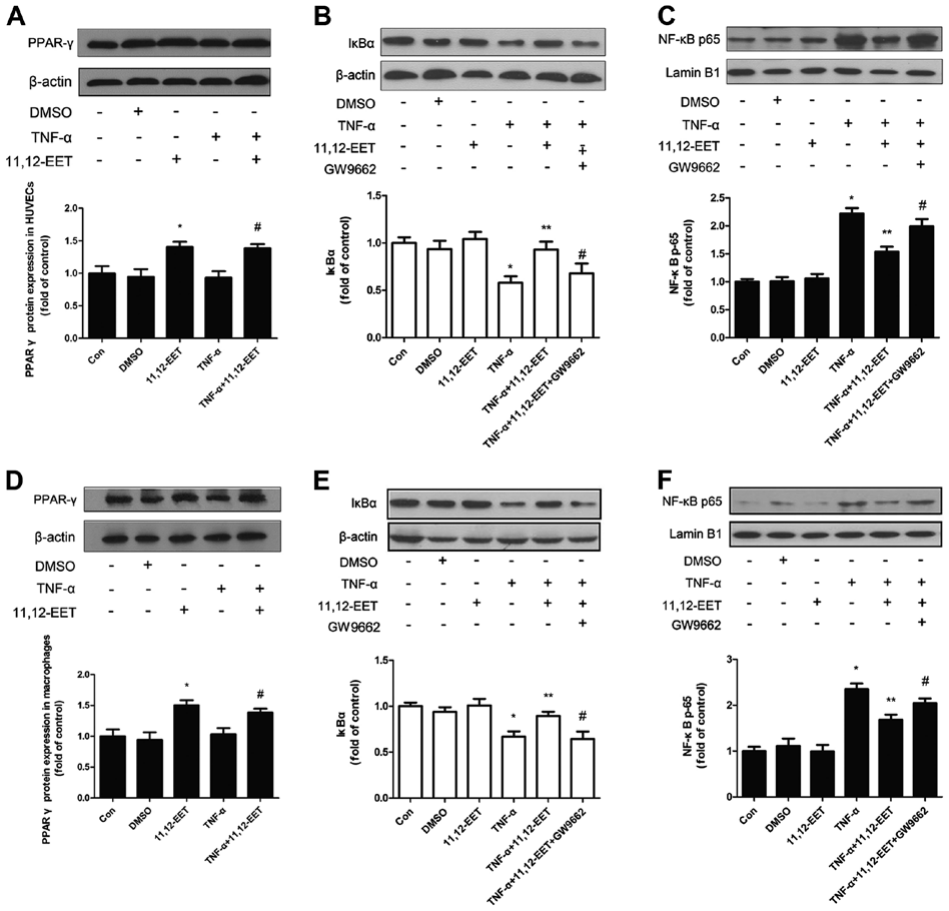
Exogenous CYP2C8-derived EETs inhibited TNF-α induced expression of NF-κB in HUVECs and macrophages. (A) PPARγ increased under the administration of 11,12-EET in HUVECs (^*^P<0.05 vs. control group; ^#^P<0.05 vs. TNF-α+EET group). (B and C) Primary cultures of HUVECs were pre-treated with EETs (1 μM) and GW9662 (1 μM) for 60 min and stimulated with TNF-α (10 ng/ml) for 24 h. (^*^P<0.05 vs. control; ^**^P<0.05 vs. TNF-α treated control; ^#^P<0.05 vs. TNF-α+EET group). (D) PPARγ increased under the administration of 11,12-EET in macrophages (^*^P<0.05 vs. control group; ^#^P<0.05 vs. TNF-α+EET group). (E and F) Primary cultures of macrophages were pre-treated with EETs (1 μM) and GW9662 (1 μM) for 60 min and stimulated with TNF-α (10 ng/ml) for 24 h. (^*^P<0.05 vs. control; ^**^P<0.05 vs. TNF-α treated control; ^#^P<0.05 vs. TNF-α+EET group). Data are the mean ± SEM of results from at least five independent experiments, each performed in triplicate.

To elucidate how CYP2C8 plays a role on anti-inflammation, it is necessary to examine the possible mechanism of CYP2C8-derived EET on anti-inflammation. Results of the western blot analysis revealed that 11,12-EET increased the protein expression of PPARγ, which was the possible acceptor of EETs and associated with inflammation (24) (Fig. 2A and D), both in the basal and inflammatory condition. Therefore, we hypothesized that the overexpression of CYP2C8 *in vitro* to increase EETs may prevent the development of inflammation potentially through PPARγ activation. Moreover, we assessed the vital transcription factor NF-κB and the conclusion was consistent in HUVECs and macrophages. IκBα expression was decreased under the stimulation of TNF-α, which could be reversed by 11,12-EET (Fig. 2B and E). The NF-κB subunit p-65 expression was opposite to the effect of IκBα (Fig. 2C and F). Notably, after adding PPARγ-specific inhibitor GW9662, the beneficial effects caused by exogenous 11,12-EET were attenuated, indicating that 11,12-EET may be involved through the PPARγ pathway in blocking the activation of NF-κB.

### 11,12-EET decreased TNF-α induced intracellular ROS production

**Figure 3:**
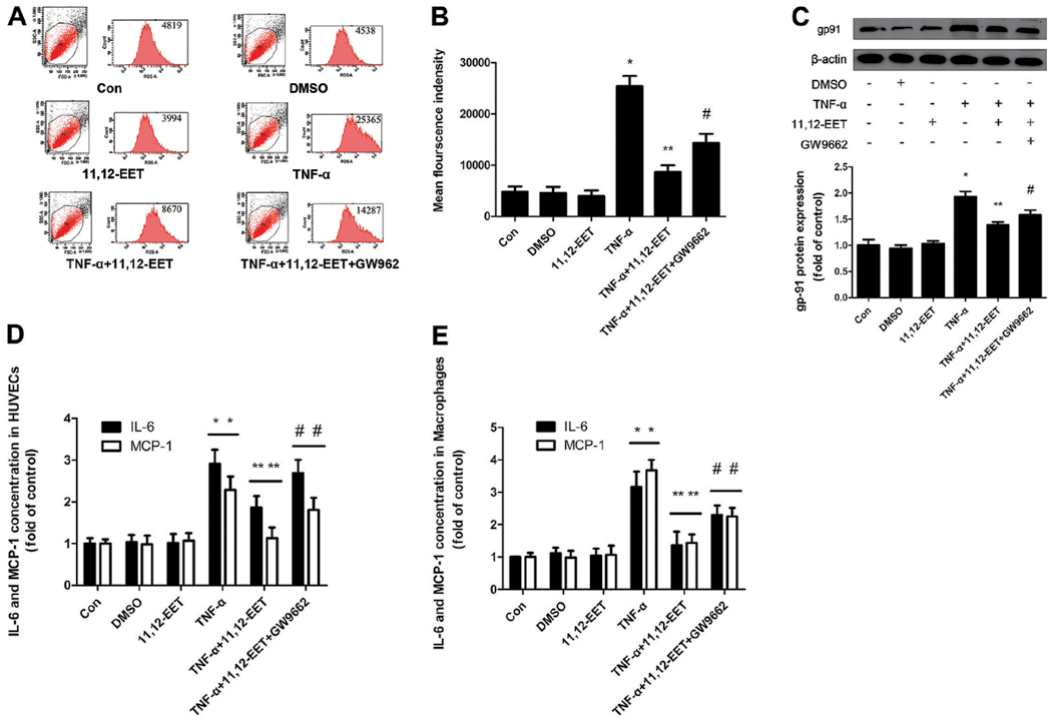
Exogenous CYP2C8-derived EETs decreases TNF-α-induced ROS production. (A) Primary cultures of HUVECs were pre-treated with EETs (1 μM) and GW9662 (1 μM) for 60 min and stimulated with TNF-α (10 ng/ml) for 24 h. Cells were stained with HB_2B_DCF-DA. ROS production was measured by a fluorescence-activated cell sorter. (B) Quantification of the mean fluorescence intensity in different groups of cells. (C) Western blot analysis revealed that gp-91 was upregulated following stimulation with TNF-α and decreased under the influence of 11,12-EET. (D and E) Exogenous 11,12-EET decreased IL-6 and MCP-1 in HUVECs and macrophages, respectively. Values are the mean ± SEM of five experiments. (^*^P<0.05 vs. control; ^**^P<0.05 vs. TNF-α treated control; ^#^P<0.05 vs. TNF-α+EET group).

Mounting evidence has indicated that the induction of ROS was necessary for the development of atherosclerosis and EETs possessed the ability of anti-atherosclerosis. To confirm the beneficial effects of 11,12-EET on atherosclerosis, intracellular ROS production induced by TNF-α in HUVECs were measured. The results showed that 11,12-EET decreased the ROS production induced by TNF-α and suppressed the TNF-α-induced mean fluorescence intensity of the dye to a level that was comparable with that of the control. However, following the addition of the PPARγ-specific inhibitor GW9662, the anti-oxidative effect caused by 11,12-EET was also attenuated (Fig. 3A and B). Results of the western blot analysis revealed that the ROS-associated NAD(P)H subunit gp-91 was increased almost 2-fold compared with the control when stimulated with TNF-α. However, this effect disappeared following the addition of 11,12-EET (Fig. 3C). Therefore, 11,12-EET performed the coincident anti-inflammatory effect through the exogenous administration and *CYP2C8* gene transfection. 11,12-EET pre-incubation reduced the expression of IL-6 and MCP-1 induced by TNF-α in HUVECs and macrophages (Fig. 3D and E). Moreover, the PPARγ-specific inhibitor GW9662 partially eliminated the beneficial effects of CYP2C8 transfection or the exogenous supply with 11,12-EET. NF-κB was one of the major transcription factors regulating the TNF-α-induced expression of inflammatory biomarkers in HUVECs and macrophages, while ROS was crucial in inflammation. Thus, the CYP2C8/EET/PPARγ/ROS/NF-κB pathway may regulate the TNF-α induced inflammatory cytokine expression in HUVECs and macrophages.

### CYP2C8-derived EETs improve endothelial function through the upregulation of eNOS

**Figure 4:**
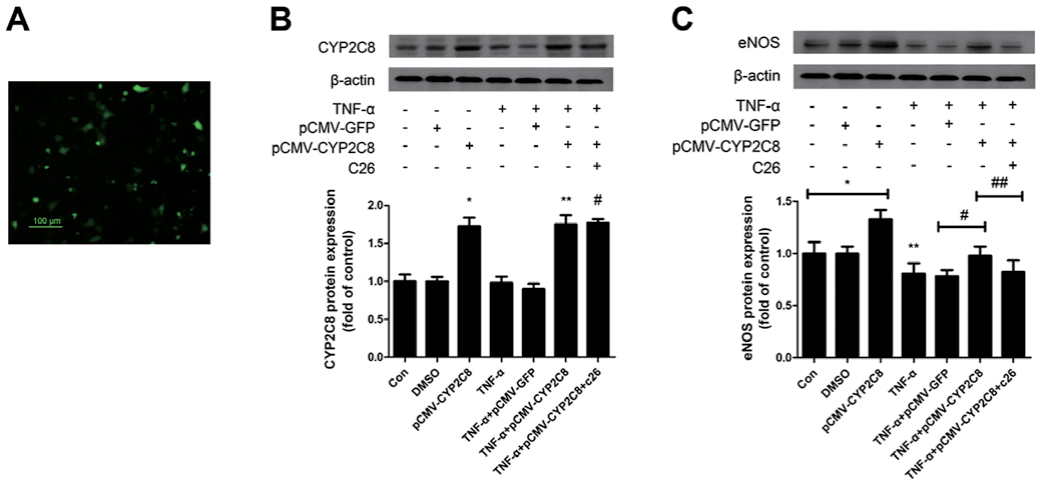
CYP2C8-derived EETs improve endothelial function through the upregulation of eNOS. (A) pCMV-CYP2C8 transfection led to a substantial expression of CYP2C8 in HUVECs. (B) Western blot analysis revealed the CYP2C8 protein expression. (C) The expression of eNOS in HUVECs. Data are the mean ± SEM of results from at least five independent experiments, each performed in duplicate (^*^P<0.05 vs. control; ^**^P<0.05 vs. TNF-α treated control; ^#^P<0.05 vs. TNF-α+pCMV-CYP2C8 group).

In addition, eNOS catalyzed the formation of NO characterized as an anti-atherosclerosis effect. To examine how CYP2C8-derived EETs affect the eNOS expression, CYP2C8 transfection was performed. pCMV-CYP2C8 transfection led to a substantial expression of CYP2C8 in HUVECs. Moreover, western blot analysis revealed a CYP2C8 protein overexpression in HUVECs (Fig. 4A and B) and eNOS was upregulated by CYP2C8 overexpression, which was decreased significantly under the stimulation of TNF-α (Fig. 4C). This effect suggested that CYP2C8 overexpression could increase the eNOS protein expression, which contributed to improving endothelial function and anti-atherosclerosis.

### CYP2C8-derived EETs increased the migration of VSMCs

**Figure 5:**
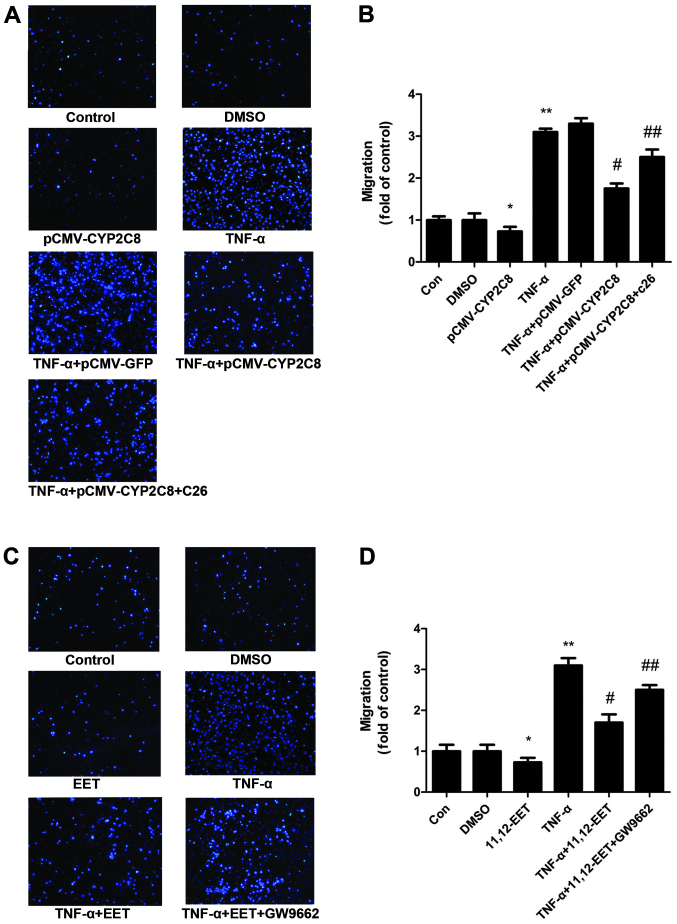
CYP2C8-derived EETs increased migration of VSMCs. (A and B) VSMCs were transfected with pCMV-CYP2C8 and pCMV-GFP and then stimulated with TNF-α. The images show optical fields at different treatments (magnification, ×200). (C and D) VSMCs were pre-incubated with EETs, GW9662 for 1 h and stimulated with or without TNF-α for 8 h. The images show optical fields at different treatments (magnification, ×200). Migration ability was quantified by the migration cell number at 8 h. The nucleus of VSMCs were stained with DAPI. Data are shown as the mean ± SEM (n=5). VSMCs were photographed and analysed by microscopy. The VSMCs were counted with Image-Pro Plus 5.0.2. Data are the mean ± SEM of results from at least three independent experiments, each performed in duplicate. (^*^P<0.01 vs. control; ^**^P<0.01 vs. TNF-α treated control; ^#^P<0.001 vs. TNF-α+EET group).

To assess the effect of CYP2C8-derived EETs on VSMC migration, we applied the Transwell migration model. pCMV-CYP2C8 transfection led to an abundant expression of CYP2C8 in VSMCs (Fig. 5A and B). The Transwell results showed that TNF-α increased the ability of the migration of VSMCs significantly. Additionally, EETs reversed the increased migration by TNF-α. TNF-α+EET+GW9662 also exerted a similar effect as TNF-α on migration (Fig. 5C and D). These results indicated that CYP2C8-derived EETs possess an anti-TNF-α effect on cell migration and may play a role through the PPARγ pathways.

## Discussion

The present study has demonstrated that CYP2C8-derived EETs significantly suppressed TNF-α-induced intracellular ROS formation and the redox-sensitive NF-κB activation via the suppression of IκB degradation and phosphorylation. We investigated *CYP2C8* gene-alleviated vascular inflammation through the CYP2C8/EET/PPARγ/ROS/NF-κB signaling pathway and improved endothelial function through the upregulation of eNOS and it may ameliorate the migration of VSMCs. These results have demonstrated that CYP2C8-derived EETs exerted antivascular inflammatory and anti-oxidative effects and it may be useful in the prevention and treatment of vascular inflammatory diseases.

Our previous study indicated that CYP2C8 exerted a protective effect on atherosclerosis induced by a western-type diet in APOEKO^+/−^CYP2C8Tg^+/−^ and CYP2C8Tg^+/−^ mice (25). The formation and area of atherosclerosis plaque was decreased significantly in teh aortic artery in the *CYP2C8* gene overexpression group. To the best of our knowledge, the present study provided the first evidence that CYP2C8-derived EETs suppressed TNF-α-induced inflammation through the suppression of NF-κB activation and ROS in HUVECs. Thus, we revealed that CYP2C8-derived EETs prevented the early pathogenesis of atherosclerosis by modulating vascular inflammation and ROS.

The production of intercellular ROS induced by cytokines such as TNF-α has been suggested in the activation of NF-κB and expression of inflammatory biomarkers (26–28). In the present study, TNF-α increased intercellular ROS production and we confirmed that CYP2C8-derived EETs decreased ROS generation in TNF-α-stimulated HUVECs. Thus, the inhibition of ROS generation by CYP2C8-derived EETs may contribute to the inhibition NF-κB activation and expression of inflammatory biomarkers.

To identify the molecular mechanism by which CYP2C8-derived EETs exerted its anti-inflammatory effect on TNF-α-stimulated endothelial cells and macrophage, we examined the activation of NF-κB, one of the major transcription factors regulating the TNF-α-induced expression of inflammatory biomarkers in endothelial cells and macrophage (29,30). NF-κB is present in the cytosol as a heterodimer composed of p50 and p65 subunits bound to the inhibitor protein IκB family in unstimulated cells. Following stimulation with cytokine, the IκB proteins are phosphorylated and degraded, which allows NF-κB to translocate into the nucleus and initiates gene transcription (31). Our present results show that CYP2C8-derived EETs significantly suppressed TNF-α-induced IκBα degradation. We also observed that CYP2C8-derived EETs inhibited the TNF-α-induced phosphorylation of NF-κB p65 at serine 536 and nuclear translocation of NF-κB p65. Furthermore, the activation of NF-κB has been associated with the transcription factor PPARγ (peroxisome proliferator-activated receptor γ) (32–34). PPARγ is a ligand-activated nuclear receptor, binding with the PPAR response element of the target gene promoter, that is involved in the transcription of the related gene (35,36). We also confirmed that CYP2C8-derived EETs decreased the levels of inflammatory factors such as MCP-1 and IL-6 in HUVECs and macrophage. Thus, our results suggest that CYP2C8-derived EETs attenuated the TNF-α-induced NF-κB activation that would be critical for the timely inhibition of inflammatory mediator expression.

Additionally, results of this study have demonstrated that CYP2C8-derived EETs improved endothelial function through the upregulation of eNOS. These results were consistent with those of a previous study (37). eNOS catalyzes the formation of NO, thus it may be characterized as exerting an anti-atherosclerotic effect.

Moreover, we confirm that CYP2C8-derived EETs are involved in the migration of VSMCs. In our experiments TNF-α increased the migration of VSMCs significantly and CYP2C8-derived EETs decreased their migration. As previous studies have demonstrated that the migration of VSMCs was associated with the formation of atherosclerosis (38–41), the decrease of the migration of VSMCs by CYP2C8-derived EETs may contribute to the improvement of atherosclerosis.

In conclusion, vascular inflammation induced by cytokine occurs early in the development of atherosclerosis and leads to endothelial dysfunction. Thus, these data have provided insight on the actions of CYP2C8-derived EETs and its potential benefits on preventing inflammatory diseases such as atherosclerosis.

## Abbreviations

CYP2C8: cytochrome P450 enzymes 2C8
EET: epoxyeicosatrienoic acid
ROS: reactive oxygen species
HUVEC: human umbilical vein endothelial cell
VSMC: vascular smooth muscle cell
NF-κB: nuclear factor κB
IκBα: inhibitor of NF-κBα
IL: interleukin
MCP-1: monocyte chemotactic protein-1
PPARγ: peroxisome proliferator-activated receptor γ
C26: compound 26
